# Sustained Release of Phosphates From Hydrogel Nanoparticles Suppresses Bacterial Collagenase and Biofilm Formation *in vitro*

**DOI:** 10.3389/fbioe.2019.00153

**Published:** 2019-06-26

**Authors:** Dylan Nichols, Marja B. Pimentel, Fernando T. P. Borges, Sanjiv K. Hyoju, Fouad Teymour, Seok Hoon Hong, Olga Y. Zaborina, John C. Alverdy, Georgia Papavasiliou

**Affiliations:** ^1^Department of Biomedical Engineering, Illinois Institute of Technology, Chicago, IL, United States; ^2^Department of Chemical and Biological Engineering, Illinois Institute of Technology, Chicago, IL, United States; ^3^Department of Surgery, University of Chicago, Chicago, IL, United States

**Keywords:** polyphosphate, nanoparticles, hydrogel, microbes, collagenase, intestinal epithelium, biofilm, monophosphate

## Abstract

Intestinal disease or surgical intervention results in local changes in tissue and host-derived factors triggering bacterial virulence. A key phenotype involved in impaired tissue healing is increased bacterial collagenase expression which degrades intestinal collagen. Antibiotic administration is ineffective in addressing this issue as it inadvertently eliminates normal flora while allowing pathogenic bacteria to “bloom” and acquire antibiotic resistance. Compounds that could attenuate collagenase production while allowing commensal bacteria to proliferate normally would offer major advantages without the risk of the emergence of resistance. We have previously shown that intestinal phosphate depletion in the surgically stressed host is a major cue that triggers *P. aeruginosa* virulence which is suppressed under phosphate abundant conditions. Recent findings indicate that orally administered polyphosphate, hexametaphosphate, (PPi) suppresses collagenase, and biofilm production of *P. aeruginosa* and *S. marcescens* in animal models of intestinal injury but does not attenuate *E. faecalis* induced collagenolytic activity (Hyoju et al., 2017). Systemic administration of phosphates, however, is susceptible to rapid clearance. Given the diversity of collagenase producing bacteria and the variation of phosphate metabolism among microbial species, a combination therapy involving different phosphate compounds may be required to attenuate pathogenic phenotypes. To address these barriers, we present a drug delivery approach for sustained release of phosphates from poly(ethylene) glycol (PEG) hydrogel nanoparticles. The efficacy of monophosphate (Pi)- and PPi-loaded NPs (NP-Pi and NP-PPi, respectively) and a combination treatment (NP-Pi + NP-PPi) in mitigating collagenase and biofilm production of gram-positive and gram-negative pathogens expressing high collagenolytic activity was investigated. NP-PPi was found to significantly decrease collagenase and biofilm production of *S. marcescens* and *P. aeruginosa*. Treatment with either NP-Pi or NP-Pi + NP-PPi resulted in more prominent decreases in *E. faecalis* collagenase compared to NP-PPi alone. The combination treatment was also found to significantly reduce *P. aeruginosa* collagenase production. Finally, significant attenuation in biofilm dispersal was observed with NP-PPi or NP-Pi + NP-PPi treatment across all test pathogens. These findings suggest that sustained release of different forms of phosphate confers protection against gram-positive and gram-negative pathogens, thereby providing a promising treatment to attenuate expression of tissue-disruptive bacterial phenotypes without eradicating protective flora over the course of intestinal healing.

## Introduction

The intestinal tract routinely undergoes a variety of injuries due to disease or direct surgical manipulation. The process by which successful repair and return of function occurs following these insults is highly dependent on the composition and function of the intestinal microbiota. Consequently, wound healing, and repair of the intestinal tract is a complex process due to the presence of intestinal microbiota, which can either enhance or severely impair the healing process. For example, severe persistent inflammation in diseases such as ulcerative colitis and Crohn's disease (i.e., inflammatory bowel disease) are recognized to be due to loss of the normal microbiome and its replacement by pathogens. In addition, iatrogenic injury, such as that which occurs when gastroenterologists remove intestinal tumors via endoscopy or major surgical resection, may also disrupt the normal microbiome leaving a large wound to heal in the presence of highly pathogenic bacteria. These curative surgical interventions are often complicated by excessive scar formation, stricture, stenosis, or grossly inadequate healing. In extreme cases, this can lead to perforation of the intestine, resulting in peritonitis and sepsis.

Previous studies indicate that certain bacteria (i.e., *E. faecalis, S. marcescens, P. aeruginosa*) are capable of secreting elevated levels of collagenase, resulting in collagen degradation (Zaborin et al., 2009, 2012; Shogan et al., 2015). The presence of this phenotype in intestinal tissue leads to excessive scar formation and healing impairment. In our previous work we identified that *E. faecalis*, a normal inhabitant of the intestinal microbiota, may be provoked to express enhanced collagenase in the gut during surgical injury, leading to major post-operative complications including anastomotic leak (Shogan et al., 2015). Using a clinically relevant mouse model of anastomotic leak, we demonstrated that exposure to pre-operative radiation followed by distal colon resection and intestinal inoculation with *P. aeruginosa*, a pathogen commonly found in the radiated intestine, results in significant incidence of leak as compared to radiated tissues alone (Valuckaite et al., 2009). *P. aeruginosa* strains retrieved from leaking anastomotic tissues were also shown to shift to a destructive phenotype (increased levels of pyocyanin and collagenase) (Olivas et al., 2012). Additional problematic pathogens such as *S. marcescens* also produce collagenases to disrupt intestinal healing (Olivas et al., 2012; Hyoju et al., 2017).

A common approach to address these issues is administration of antibiotics which indiscriminately disrupts normal intestinal microflora which have been shown to be beneficial to healing (Carlet, 2012). Clinical studies indicate that patients remain colonized by strains of *E. faecalis* and *P. aeruginosa* for as long as 1 week after surgery even after receiving oral and intravenous antibiotic administration (Ohigashi et al., 2013). This may be due, in part, to their multi-drug resistant nature as the promiscuous use of antibiotics remains a problem (Buffie and Pamer, 2013). Thus, there is an urgent need for development of non-antibiotic compounds that do not disrupt the normal intestinal microbial community structure. Normal flora protect against overgrowth and invasion of pathogens by a process of competitive exclusion and by suppressing key pathogenic traits among bacteria. One such approach is to create a nutrient rich microenvironment at the site of injury to suppress virulence expression.

Previously, we have demonstrated that phosphate-based therapy is a promising therapeutic approach for suppression of virulent expressing phenotypes since inorganic phosphate abundance is a key factor involved in attenuation of quorum sensing and global virulence (Zaborin et al., 2009). Furthermore, we have demonstrated that depletion of extracellular phosphate occurs in the intestinal tract following surgical injury triggering bacterial virulence (Zaborin et al., 2009, 2012). As a result of extracellular phosphate depletion, pathogens scavenge phosphate from host tissues, leading to disruption of intestinal mucus. Our *in vivo* and *in vitro* findings indicate that replenishment of monophosphate (Pi) in the intestinal epithelium via oral administration prevents virulence expression while maintaining bacterial survival (Zaborin et al., 2009). Polyphosphate, a polymer of phosphate residues linked by high-energy phosphoanhydride bonds as in ATP, also plays a key mechanistic role in survival and virulence across a broad range of bacteria (Rao et al., 2009). Our recent findings indicate free PPi decreases *in vitro* collagenase and biofilm production of gram-negative *P. aeruginosa* and *S. marcescens* and that oral administration of PPi prevents anastomotic leak in mice subjected to intestinal injury and inoculation with these pathogens (Hyoju et al., 2017). Conversely, PPi treatment did not demonstrate significant collagenase attenuation of gram-positive *E. faecalis* or prevent *E. faecalis*-induced anastomotic leak (Hyoju et al., 2017). Given the diversity of bacteria that produce collagenase and the variation in phosphate metabolism among microbial species, a combination therapy involving different phosphate compounds may be required to attenuate the expression of pathogenic phenotypes. In addition, oral administration of inorganic phosphate is prone to loss of bioavailability in the colon, a common site of surgery to remove cancerous tumors. Inorganic phosphate is rapidly absorbed in the small intestine, and as a result, high concentrations of orally administered phosphates are required to increase phosphate concentration which may impair kidney function.

To address these barriers, we present a drug delivery approach that provides sustained release of phosphates from hydrogel nanoparticles to attenuate collagenase and biofilm production across gram-negative and gram-positive pathogens. Successful *in vitro* demonstration of this approach holds promise for *in vivo* replenishment of phosphate levels in intestinal tissue. In previous work we developed a novel process to produce crosslinked hydrogel nanoparticles of poly(ethylene) glycol (NP) for loading of hydrophilic therapeutics, including phosphates, enabling their sustained release (Vadlamudi et al., 2019). We have shown that this process results in the production of phosphate as well as polyphosphate loaded NPs (NP-Pi and NP-PPi, respectively) and in sustained release of Pi and PPi (Yin et al., 2017). Importantly, our published findings indicate that NP-PPi attenuate *P. aeruginosa* pyoverdine production in a dose-dependent manner and significantly decrease pyocyanin production while maintaining bacterial survival (Yin et al., 2017). The goal of this study is to determine the effectiveness of NP-PPi and a combination treatment of NP-Pi and NP-PPi (NP-Pi + NP-PPi) for suppression of collagenase and biofilm across gram-positive and gram-negative pathogens. In this work we evaluate the minimal inhibitory concentration of the phosphate-loaded nanoparticle formulations and screen their efficacy in suppressing *in vitro* collagenase and biofilm production for three key pathogens (*P. aeruginosa, S. marcescens*, and *E. faecalis)* identified to express high collagenolytic activity and to induce anastomotic leak in both rodents and humans.

## Materials and Methods

### Materials

Poly(ethylene glycol) diacrylate (PEGDA, MW = 575 Da), N-vinyl pyrrolidone (NVP), potassium phosphate monobasic (phosphate, Pi), sodium hexametaphosphate (polyphosphate, PPi), potassium persulfate (KPS), sorbitan monooleate (SPAN80), PEG (20) sorbitan monolaurate (TWEEN20), cyclohexane, and acetone were purchased from Sigma-Aldrich (St. Louis, MO, USA). Ultra-high purity (UHP) nitrogen was purchased from Airgas (Radnor, PA, USA).

Monomethyl ether hydroquinone (MEHQ) inhibitor (400–600 ppm) was removed from the PEGDA monomer by passing the solution through a prepacked column (Sigma Aldrich). Sodium hydroxide inhibitor was removed from the NVP monomer by passing the solution through a 0.2 μm syringe filter (Millipore, Burlington, MA, USA). All other materials were used as received.

### Nanoparticle Synthesis, Purification, Phosphate, and Polyphosphate Loading

Hydrogel nanoparticles were created using an inverse phase miniemulsion polymerization process we have formerly developed for encapsulation of monophosphate (i.e., potassium monobasic phosphate) but with slight modifications as described herein (Yin et al., 2017; Vadlamudi et al., 2019). The nanoparticle synthesis process requires the formation of a lipophobe, typically an inorganic salt (i.e., phosphate), for the prevention of Ostwald ripening (Vadlamudi et al., 2019). To achieve sufficient loading of polyphosphate (PPi) within the hydrogel nanoparticles, potassium monobasic phosphate (monophosphate) loaded nanoparticles were first created using inverse phase miniemulsion polymerization, purified of excess reactants, rinsed to remove monophosphate, and post-loaded with PPi (as described below). This enabled loading of a desired PPi concentration post nanoparticle synthesis. Monophosphate (Pi)-loaded nanoparticles (NP-Pi) were also synthesized as previously described (Yin et al., 2017; Vadlamudi et al., 2019). The polymerization reaction was carried out in an inverse miniemulsion created through emulsification of an aqueous phase precursor into an organic phase (Vadlamudi et al., 2019). The aqueous phase consisted of a precursor solution containing a PEG diacrylate (PEGDA) crosslinker, N-vinyl pyrrolidone (NVP), sorbitan monolaurate (TWEEN 20) as the water-soluble surfactant, potassium persulfate (KPS) as the thermal initiator and potassium monobasic phosphate (monophosphate) as the osmotic pressure agent in deionized (DI) water. The total concentration of cross-linkable double bonds in the precursor was fixed at 1.36 M and the initiator concentration was set as 1.5 % molar ratio of initiator to reactive double bonds. The organic phase contained cyclohexane and sorbitan monooleate (SPAN 80) as the organic soluble co-surfactant.

The inverse miniemulsion was generated using a two-step homogenization process. The organic phase was chilled in an ice bath and then homogenized using a rotor-stator homogenizer (10 mm saw-tooth generator, IPRO 250, Pro-Scientific) at 20,000 rpm. The aqueous phase was added drop-wise to the organic phase and homogenization continued for 15 min. The resulting emulsion was exposed to high energy sonication using an ultrasonic horn (SONICS Vibracell VCX 750 Watts) at 90% amplitude for five pulses of 1 min each. The stable inverse miniemulsion was transferred to a sealed glass batch reactor, kept under a nitrogen blanket and continuously sparged with pure nitrogen for 1 h. When sparging was stopped the reactor was partially immersed in an oil bath set to achieve a reaction temperature of 56± 0.5°C. The reaction proceeded at this temperature for 4.5 h after which the resulting stable nanoparticle suspension was removed from the reactor and allowed to cool in a glass jar at room temperature.

PEG hydrogel nanoparticles were precipitated from the colloidal suspension by the addition of 70–80 mL of acetone. Agglomerated particles were allowed to settle overnight at 4°C after which two layers were formed, a white bottom layer and yellowish supernatant. The supernatant was removed by decantation followed by addition of 120 mL of fresh acetone and re-suspension by sonication at 90% amplitude. The suspension was centrifuged at 3,220 g at 4°C for 90 min followed by removal of the supernatant and replacement with fresh acetone. This sonication and centrifugation cycle was completed twice more to ensure removal of residual cyclohexane and surfactant. Rinsed nanoparticle pellets were dried under vacuum for 48 h. Dried nanoparticle agglomerates were transferred to a mortar and crushed with a pestle into a fine powder, yielding monophosphate loaded nanoparticles (NP-Pi).

To remove encapsulated monophosphate, nanoparticles were rinsed with DI water, sonicated, swollen in DI water for 2 h, followed by centrifugation at 3,220 g and 4°C for 30 min and removal of supernatant. This process was repeated eight times to ensure complete removal of the monophosphate from the nanoparticles (confirmed by measuring the amount of phosphate release using the molybdenum blue method as described below). Fully rinsed nanoparticles devoid of phosphate (blank nanoparticles) were used as controls in all experiments.

Post-loading of heat treated PPi (56°C for 4.5 h) (Yin et al., 2017) into blank NPs was achieved by suspending 1.5 grams of blank NPs in 40 mL of a 150 mg/mL solution of sodium hexametaphosphate (PPi). The solution was sonicated at 15 % amplitude for 5 s and stored at 37°C for 72 h. The nanoparticles were then centrifuged at 3,220 g and 4°C for 30 min and the supernatant was removed. To remove excess PPi from the particle surface, nanoparticles were rinsed with 5 mL DI water, centrifuged, and the supernatant removed. Polyphosphate loaded nanoparticles (NP-PPi) were frozen at −80°C, lyophilized, crushed with a pestle into a fine powder, and stored dry at room temperature until use.

### Nanoparticle Characterization

Three types of nanoparticles were used in this study: sodium monophosphate loaded NPs (NP-Pi), blank NPs (formed by rinsing NP-Pi to remove Pi), and sodium hexametaphosphate-loaded nanoparticles (NP-PPi), formed from blank NPs subsequently post-loaded with PPi. All nanoparticles were synthesized as three separate batches and particle size distribution, diameter, ζ–potential, and swelling ratio were obtained as an average from all batches. Particle size and ζ–potential measurements were performed using a 0.1 mg/mL nanoparticle concentration. Particle size distribution was quantified using a nanosizer (NanoSight LM10, Malvern, UK) equipped with nanoparticle tracking analysis software (NanoTracking v 3.0). Zeta (ζ-) potential was determined using dynamic light scattering (DLS) (Zetasizer Nano ZS, Malvern, UK) with measurements made in a 10 mM NaCl solution. The mass swelling ratio of hydrogel nanoparticles was determined by gravimetric analysis and calculated from the ratio of the swollen (in PBS) to dry nanoparticle weights. All values were obtained as averages of at least three independent measurements. SEM imaging was used to visualize the shape and formation of blank (NP) and PPi-loaded nanoparticles (NP-PPi) in the dehydrated (dried) state. Briefly, nanoparticles were dissolved in 99% ethyl alcohol to a concentration of 1 mg/mL. The solution was vortexed and sonicated for mixing. A metal support stub was covered with round carbon tape adhesives onto which the solution was pipetted. Stubs containing NP or NP-PPi were left under the hood until all the ethanol evaporated. Stubs were then imaged using an JEOL JSM 6701-F Field Emission Scanning Electron Microscope and images of the nanoparticles acquired.

### Quantification of Polyphosphate Release Kinetics From NP-PPi

Polyphosphate release from nanoparticles was measured via a molybdenum blue absorbance assay at 340 nm (Phosphorus Liqui-UV No. 0830, StanBio Laboratory, USA). A standard curve was created for PPi (heat treated) in tryptone yeast (TY) media using the molybdenum blue assay kit. Nanoparticles were incubated in TY media (18 mg/mL) at 37°C over 72 h. PPi released from nanoparticles was measured under near perfect sink conditions at several pre-determined times. Specifically, nanoparticles were centrifuged at 6,000 g for 3 min, followed by removal of the supernatant (1 mL) and replacement with fresh TY media. Release kinetics of Pi from NP-Pi have been reported in our prior studies (Yin et al., 2017).

### Collagenolytic Bacterial Strains

The commercially available constitutive bioluminescent *P. aeruginosa* PAO1 derivative XEN41 strain with the *luxCDABE* cassette incorporated into chromosome (Calpier Life Sciences, Inc), was used in these studies. This strain was previously confirmed in mouse model experiments (Fink et al., 2011) to display a highly collagenolytic P2 phenotype (Luong et al., 2014). *S. marcescens* ICU2-4 strain was isolated from an ICU patient at the University of Chicago (Zaborin et al., 2014) and was characterized as collagenolytic (Hyoju et al., 2017). A bioluminescent strain ICU2-4*lum* for *S. marcescens* was created by transformation with the pAKlux2 plasmid containing the *luxCDABE* luciferase cassette (Addgene, Ref #14080) (Karsi and Lawrence, 2007). The antibiotic resistant strain *E. faecalis* E61 was isolated from rat anastomotic tissue and was characterized as collagenolytic (Shakhsheer et al., 2016). All bacterial strains were stored in 10 % glycerol stock at −80°C. Cells from frozen stocks were plated onto tryptone yeast (TY) plates and grown overnight at 37°C. An inoculation loop of plated bacteria was transferred to culture tubes containing 2 mL of TY media that was subsequently cultured overnight at 37°C. In all experiments, bacterial strains were utilized from these liquid cultures and diluted 1:100 in fresh TY media.

### Minimal Inhibitory Concentration of NP-Pi, NP-PPi, and Mixtures of NP-Pi + NP-PPi

The minimal inhibitory concentration (MIC) of nanoparticle formulations [concentration range between 1 and 10% (w/volume)] was determined for the three pathogens. In the case of gram-negative pathogens, an MIC screening was conducted for NP-PPi and a mixture of NP-Pi + NP-PPi since our prior findings indicated that monophosphate (Pi) treatment (free Pi or NP-Pi) does not attenuate collagenase production for these pathogens (data not shown). The total concentration of nanoparticles used was kept constant among all treatments. In the case of *E. faecalis*, an additional treatment group, NP-Pi, was also explored as monophosphate was previously shown to attenuate its collagenolytic activity. Six different NP treatment concentrations were tested [1, 2, 4, 6, 8, and 10% (w/v)] for each pathogen in addition to the no treatment control. To determine the MIC values of each formulation for each pathogen, overnight cultures were diluted 1:100 in TY medium, inoculated with the specified treatment concentration and incubated at 37°C. CFUs were then quantified for each group and the MIC value was determined based on the lowest nanoparticle concentration at which statistical inhibition in growth was noted.

### Evaluation of Bacterial Collagenolytic Activity

Bacterial collagenolytic activity was evaluated in the absence and presence of monophosphate and polyphosphate treatment. Treatment groups included free PPi (heat-treated), blank nanoparticles (NP), NP-Pi, NP-PPi, and mixtures of NP-Pi and NP-PPi (NP-Pi + NP-PPi). Collagenase levels were quantified using a collagenase assay kit (DQ™ gelatin from pig skin, fluorescein conjugate, Invitrogen, Eugene, OR). Bacterial strains were grown overnight in liquid TY media and then diluted 100-fold. A volume of 190 μl of diluted bacteria in liquid TY, with or without PPi treatment, was added to 10 μl of collagen substrate (1 mg/mL; DQ gelatin from pig skin, fluorescein conjugate). The negative control consisted of 190 μl TY media with 10 μl of collagen substrate. The result of the reaction was measured at the appropriate time points with fluorescence excitation and emission at 485 and 528 nm, respectively, using a microplate reader (FL x800, Bio-Tek Instruments Inc). Values obtained for negative controls were subtracted from experimental treatment groups to account for background fluorescence. All experiments were carried out in triplicate. At each time point, the luminescence of each sample was measured and normalized to the number of bacteria in each sample.

### Bacterial Adherence/Biofilm Assay

Overnight culture of bacterial strains was diluted 1:100 in 500 μl TY media. The OD of 1:100 dilutions of overnight cultures was in the range of 0.02–0.05 (OD = 600 nm) Depending on the group (with or without nanoparticle treatment) nanoparticles were added to the bacterial solution right before being aliquoted into 96-well polystyrene plate. Plates containing 200 μl per well of this solution were incubated at 37°C for 24 h and further accessed for biofilm formation *via* crystal violet staining (Shakhsheer et al., 2016). Briefly, after incubation the medium was removed from the plates and used for quantifying CFUs for all bacterial strains. Plates were gently washed three times with DI water and allowed to dry. Two hundred μl of 0.1% crystal violet was added to each well at room temperature. After 20 min the stain was removed, and the plates gently washed three times with DI water. After drying, 200 μl of ethanol (95%) was added to solubilize the crystal violet stained in each well and the absorbance was then measured at 590 nm and normalized to growth.

### Quantification of Bacterial Growth

Optical density measurements are commonly used to normalize collagenase and adherence values to the number of bacteria in each sample; however, the presence of nanoparticles in culture interferes with the accuracy of optical density measurements for quantifying bacterial growth. To address this issue, we used bioluminescent bacterial strains for *P. aeruginosa* and *S. marcescens* as described above to quantify growth via luminescence. Comparisons of luminescence to CFUs were made to ensure referenceable values for bacterial growth. This was achieved by evaluating luminescence and plating for CFUs at different time points. A standard curve of luminescence and CFUs was then created allowing for quantification of an “effective” CFU value for each observed luminescent value. This technique was used for bioluminescent gram-negative bacterial strains. Standard curves for luminescence and CFUs are provided as supplementary data (Figures S1, S2). In the case of *E. faecalis*, bacterial growth was measured by counting colony forming units (CFUs).

### Statistical Analysis

Each experiment was performed in triplicate unless otherwise noted. Quantitative results are presented as the mean, plus, and minus the standard deviation. Statistical comparisons were performed using analysis of variance (ANOVA) followed by a Holm-Sidak test for comparisons of data pairs. Statistical significance was considered for *p* < 0.05.

## Results and Discussion

### Nanoparticle Characterization

**Figure 1:**
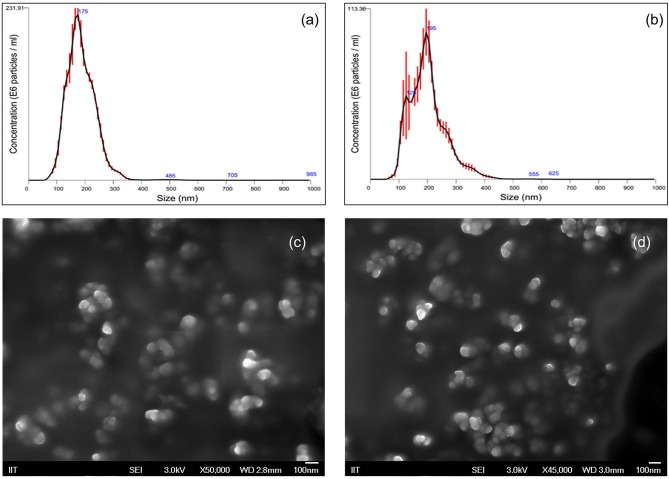
Nanoparticle Characterization using Nanoparticle Tracking Analysis and Scanning Electron Microscopy. Particle size distributions for **(a)** blank NP (D10: 116.5 nm, D50: 170.6 nm, D90: 239.5 nm) and **(b)** NP-PPi (D10: 113.4 nm, D50: 184.2 nm, D90: 276.1 nm). Red error bars indicate ± 1 standard error of the mean from three separate nanoparticle tracking measurements. SEM representative images of **(c)** blank NPs and **(d)** NP-PPi in the dehydrated state.

The *in vitro* effectiveness of phosphate-loaded NPs in attenuating collagenase and biofilm formation was evaluated against a control of blank nanoparticles (NPs) devoid of Pi or PPi. Since variations in particle physical properties have been shown to influence cell-nanoparticle interactions, and to discern whether the observed *in vitro* findings are mainly attributable to Pi or PPi treatment, we first characterized the physicochemical properties of blank NPs, NP-Pi, and NP post-loaded with PPi (NP-PPi). All nanoparticles were characterized in terms of hydrated particle size distribution, gravimetric swelling ratio (inversely related to the crosslink density of the nanoparticle network), and ζ-potential (an indicator of particle surface charge characteristics). The values of each of these properties are reported in Table 1 and represent the average of three separate nanoparticle synthesis batches for each particle type. As expected, the mean diameter was found to be similar for NP, NP-PPi and NP-Pi (181 ± 57, 197 ± 63 nm, 181 ± 57, respectively) with uniform particle size distribution as quantified by nanoparticle tracking analysis (Figures 1a,b). The swelling ratios of NP and NP-Pi (4.26 ± 0.26) and NP-PPi (4.05 ± 0.22) were similar as these particles were synthesized using identical polymerization conditions resulting in similar network structure (i.e., crosslink density) prior to post-loading with PPi. In addition, blank NPs were synthesized by first forming NP-Pi followed by particle rinsing to remove Pi. The ζ-potentials of NP-Pi and NP-PPi were found to be more negative (−17.92 ± 1.05 mV and −14.27 ± 5.34 mV, respectively) as compared to NP (−11.80 ± 5.27) which is expected due to the presence of phosphate ions in the former case. The ζ-potential of NP-Pi was slightly more negative as compared to NP-PPi which may be attributed to the increased diffusivity and accumulation of monophosphate toward the nanoparticle surface as compared to PPi. Conductivity of all NP formulations was consistently between 1.0 and 1.1 mS/cm. Collectively, the data suggest that the nanoparticles imbibe roughly four times their dry weight in aqueous solution and that they possess negative surface charge characteristics. Negative surface charge is desired for our intended application as it minimizes nanoparticle cellular uptake since the goal is to provide sustained release of phosphates extracellularly. Importantly, the data indicate no significant differences between NP, NP-Pi, and NP-PPi physicochemical properties, which allows us to attribute the observed *in vitro* responses to PPi release. SEM imaging was used to further verify the formation and visualize the shape of the resultant nanoparticles. Figures 1c,d provides representative SEM images of blank NPs (devoid of phosphate) and NP-PPi, respectively, in the dehydrated state. SEM imaging confirms similar nanoparticle morphology, shape and diameter in the presence or absence of Pi or PPi loading. A spherical particle morphology and diameter <100 nm can be discerned from each of the acquired SEM images of nanoparticles in the dehydrated state. The nanoparticle diameters are expected as to be lower than those of the fully swollen state, the latter quantified using nanoparticle tracking analysis.

**Table 1:** Particle diameter, swelling ratio, and ζ-potential for blank and PPi-loaded hydrogel nanoparticles.

	**Nanoparticle diameter (nm)**	**Mass swelling ratio**	**Zeta potential (mV)**
NP (blank)	181 ± 57	4.26 ± 0.16	−11.80 ± 5.27
NP-PPi	197 ± 63	4.05 ± 0.22	−14.27 ± 5.34
NP-Pi	181 ± 57	4.26 ± 0.16	−17.92 ± 1.05

### Release Kinetics of Polyphosphate From NP-PPi

**Figure 2:**
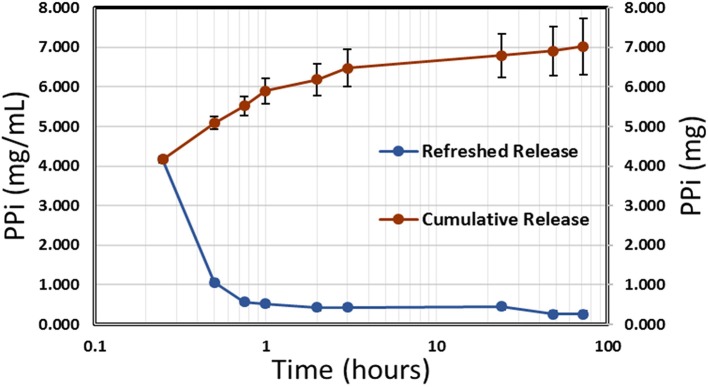
Release kinetics of PPi from NP-PPi in TY media. Refreshed release indicates single time-point measurements (left vertical axis) and cumulative release indicates the sum of all previous single measurements (right vertical axis).

Release of polyphosphate from NP-PPi was analyzed using a phosphorus absorbance assay kit (StanBio Labs, Boerne, Texas). A polyphosphate standard curve was generated for absorbance measurements with a PPi solution in tryptone yeast (TY) media, which was the media used during *in vitro* experiments with the different bacterial strains. The standard curve was linear in the range of 0.1–12.5 mg/mL PPi. Polyphosphate release from NP-PPi was measured in TY media at pre-determined times until PPi release could no longer be detected. Figure 2 displays the concentration of PPi released at specified times (refreshed release) as well as the cumulative release under near perfect sink conditions. The data shown in Figure 2 indicate an initial burst of PPi over the first hour with sustained release after that time until 72 h. The cumulative release maximum was estimated to be 7.0 mg/mL PPi which represents the total amount of PPi released from the 18 mg/mL nanoparticle sample. To determine an “equivalent free PPi concentration” to use for *in vitro* experiments, release of PPi in TY media over the 72-h time period was measured for different NP-PPi concentrations. The measured absorbance was then converted to the ratio of PPi concentration over the initial concentration of NP-PPi. These concentrations allowed us to estimate an average equivalent polyphosphate ratio of 37 ± 0.01% PPi. Figure 2 displays the PPi release for one representative batch of nanoparticles. However, two subsequent batches yielded similar release profiles and equivalent PPi concentrations. Thus, the equivalent free PPi concentration that was used in subsequent *in vitro* studies was estimated as 37% of the NP-PPi concentration. The theoretical maximum concentration of PPi within the nanoparticles was calculated as the equilibrium concentration after diffusion of the PPi post-loading solution into the nanoparticle load. This calculation yielded a maximum PPi concentration of 36% of the NP-PPi weight. Accounting for error, this would indicate a loading efficiency of nearly 100%. The release data also suggest that the network swelling ratio and crosslink density is sufficient for post-loading within and subsequently releasing PPi from the hydrogel nanoparticles.

### Effect of Free PPi on Bacterial Collagenase Activity

We have previously shown that oral administration of free PPi is effective at suppressing collagenase production and preventing anastomotic leak of gram-negative *S. marcescens* and *P. aeruginosa* (Hyoju et al., 2017). Free PPi treatment was not, however, found to be effective for *E. faecalis* (Hyoju et al., 2017). Since clinical studies have shown that patients remain colonized with pathogenic strains as long as 7 days post-surgery (Ohigashi et al., 2013), nanoparticles providing sustained release of PPi over this duration offer a promising drug delivery strategy for attenuation of collagenase activity. The ability of NP-PPi or NP-Pi + NP-PPi combinations to suppress collagenase and biofilm formation *in vitro* has not been previously explored and was evaluated for three different pathogens exhibiting high collagenolytic activity (*P. aeruginosa, S. marcescens*, and *E. faecalis*).

**Figure 3:**
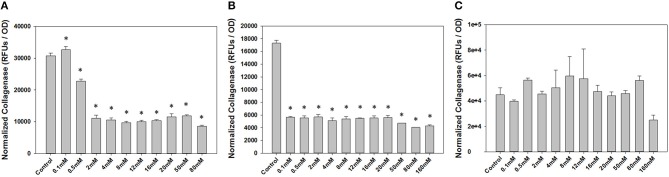
Effects of free PPi dosing on collagenase activity normalized to growth via optical density measurements for **(A)** *P. aeruginosa*, **(B)** *S. marcescens* after 8 h of incubation, and **(C)** *E. faecalis* after 16 h of incubation. Error bars indicate plus and minus the standard deviation. Asterisk {_*_} denotes significance vs. control with *p* < 0.05 considered statistically significant.

**Figure 4:**
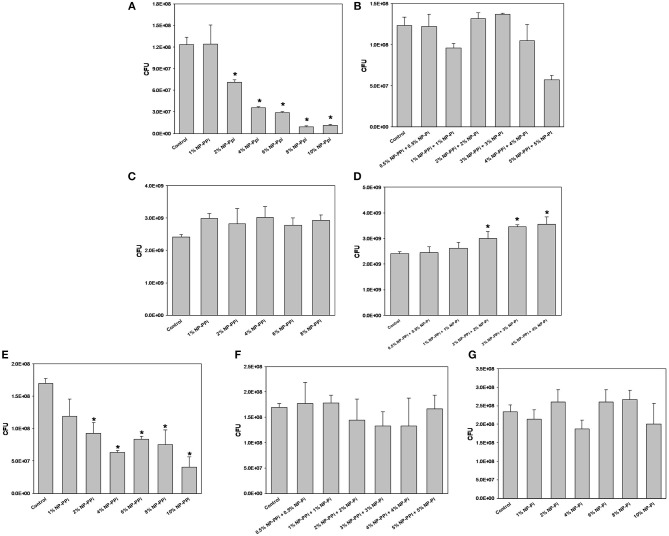
Minimal inhibitory concentration (MIC) screening of nanoparticle formulations for test pathogens. MIC of NP-PPi for **(A)** *P. aeruginosa*, **(C)** *S. marcescens, and* **(E)** *E. faecalis*. MIC of NP-Pi and NP-PPi mixtures for **(B)** *P. aeruginosa*, **(D)** *S. marcescens, and* **(F)** *E. faecalis*. MIC of NP-Pi for **(G)** *E. faecalis*. Error bars indicate plus and minus the standard deviation. Asterisk {_*_} denotes significance vs. control with *p* < 0.005 considered statistically significant.

To determine a concentration of NP-PPi to be used for *in vitro* studies, we first conducted dose-response studies of collagenase attenuation with free PPi for all three test pathogens. Concentrations of PPi tested ranged from 0.1 to 160 mM (Figure 3). This range was chosen since microorganisms can synthesize polyphosphates at concentrations as high as 200 mM (Harold, 1966) which is dramatically higher as compared to reported concentrations in animal tissue (μM concentration) (Kumble and Kornberg, 1995; Lorenz et al., 1997). Collagenase activity and optical density were also measured after 8 hours of growth for gram-negative pathogens (*P. aeruginosa* and *S. marcescens)* and after 16 h of growth for gram-positive *E. faecalis*. These two time points were chosen as we observed that 8 h of growth for both *P. aeruginosa* and *S. marcescens* and 16 h for *E. faeclais* were sufficient to “stress” the cultures under nutrient depletion. This induced “stress” can be noted by the high collagenase activity displayed at the 8- and 16-h time points. Figure 3 shows the effect of free PPi concentration on normalized collagenase levels for each pathogen. At PPi concentrations greater or equal to 2 mM, significant attenuation in *P. aeruginosa* collagenase activity is observed while PPi concentrations greater or equal to 0.1 mM were found to be effective for suppression of *S. marcescens* collagenolytic activity. No discernable response to PPi treatment was observed for *E. faecalis*, although a reduction in collagenase was noted at a PPi concentration of 160 mM. This decrease, however, was not found to be statistically significant from the no treatment control. Based on these results, 6 mM PPi was chosen as an effective dose for targeting both *P. aeruginosa* and *S. marcescens* collagenase attenuation. This target concentration was chosen because as it was greater than the minimum dose found to attenuate collagenase expression in the gram-negative test pathogens (2 mM PPi). In addition, 6 mM PPi corresponds to a concentration of 1% (weight per volume) NP-PPi which was found to be below the MIC value of the gram-negative and gram- positive test pathogens (Figure 4). A concentration of 6 mM free PPi corresponds to an equivalent concentration of 3.7 mg/mL (0.37% w/vol) which was subsequently used in culture studies.

### Bactericidal Activity of Nanoparticles

The minimum inhibitory concentrations (MIC) of phosphate-loaded nanoparticle formulations were tested within the range of 1–10% nanoparticles (w/vol). This chosen range of nanoparticle concentration also corresponds to 6–60 mM free PPi which was found to be effective at attenuating collagenase levels of the gram-negative test pathogens. Since previous studies (Hyoju et al., 2017) and the results from Figure 3C indicated that free PPi was not effective at attenuating *E. faecalis* collagenase production, we hypothesized that NP-Pi or a combination treatment of NP-Pi + NP-PPi would be more effective, the latter beneficial for attenuating collagenase across multiple pathogens. An MIC screening for NP-PPi and NP-Pi + NP-PPi was conducted in the case of *P. aeruginosa* and *S. marcescens*. The MIC value was taken as the minimum concentration that resulted in statistical significance in reduction in CFU as compared to the no treatment control. As shown in Figure 4A, the MIC for NP-PPi was 2% (w/vol) in the case of *P. aeruginosa* between the tested range of 1–10% (w/vol) NP-PPi. No bactericidal effects were observed with NP-Pi + NP-PPi treatment for *P. aeruginosa* (Figure 4B). In addition, no bactericidal effects were for NP-PPi and NP-Pi + NP-PPi treatments were found in the case of *S. marcescens* over the tested nanoparticle concentration range (Figures 4C,D). Similar to the findings obtained for *P. aeruginosa*, a bactericidal effect was noted with concentrations greater or equal to 2% (w/vol) NP-PPi for *E. faecalis* (Figure 4E). No bactericidal effects were observed in the case of the combination treatment or with NP-Pi over the 1–10% (w/vol) nanoparticle range for *E. faecalis* (Figure 4F). Thus, the MIC value for NP-PPi was taken to be ≥2% (w/vol) and a total nanoparticle concentration of 1% (w/vol) was used in all subsequent studies involving collagenase and biofilm production.

### Effect of Nanoparticle Formulations on Collagenase Activity and Growth

**Figure 5:**
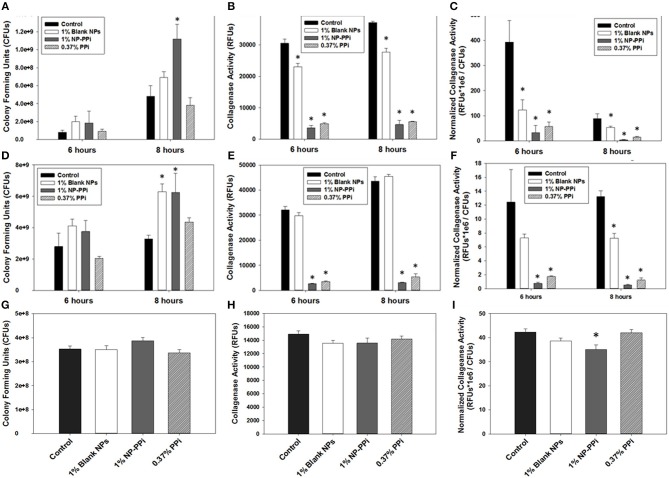
Effect of PPi formulations on collagenase attenuation. Effect of treatment formulations on growth of **(A)** *P. aeruginosa*, **(D)** *S. marcescens, and* **(G)** *E. faecalis;* on raw collagenase activity of **(B)** *P. aeruginosa*, **(E)** *S. marcescens, and* **(H)** *E. faecalis;* on normalized collagenase activity of **(C)** *P. aeruginosa*, **(F)** *S. marcescens, and* **(I)** *E. faecalis*. Error bars indicate plus and minus the standard deviation. Asterisk {_*_} denotes significance vs. control with *p* < 0.05 considered statistically significant.

Common antibiotics to prevent infection during intestinal surgery often indiscriminately eliminate the normal flora. This approach allows pathogenic bacteria to “bloom” and become antibiotic resistant. To determine the effectiveness of NP-PPi in maintaining bacterial survival, growth was quantified in the presence and absence of PPi treatment. The experimental groups included the no-treatment control, and treatment groups with blank NPs and NP-PPi (both at 1% w/vol) and equivalent free PPi (0.37% w/vol). Negative controls without nanoparticles were also included to confirm that the presence of NPs did not interfere with bioluminescence or fluorescence measurements for quantifying bacterial growth. Both blank NPs and NP-PPi were found to promote *P. aeruginosa* growth at 6 h with statistical increases at 8 h as Figure 5A. In terms of raw collagenase values, the presence of PPi and NP-PPi results in significant attenuation of collagenase activity at both 6 and 8 h (Figure 5B). A significant drop in normalized collagenase is observed for all groups at the 8-h time point as compared to the 6-h time point (Figure 5C) which is attributed to the observed increases in *P. aeruginosa* growth at 8 h (Figure 5A). Overall, an over 90% reduction in collagenase is observed as compared to the no treatment control at both time points (Figure 5C). While decreases in raw collagenase levels due to blank NP treatment are indicated, the presence of supplementation with NP-PPi and equivalent PPi result in statistical attenuation of collagenase activity of *P. aeruginosa*, with both treatments attaining at least a 6-fold decrease in normalized collagenase levels.

For *S. marcescens*, similar effects to those observed for *P. aeruginosa* occurred with increases in growth from 6 to 8 h (Figure 5D). While NP-PPi and PPi treatments significantly attenuated *S. marcescens* collagenase activity, the presence of blank NP had no effect on raw collagenase levels (Figure 5E). In terms of normalized collagenase, however, significant attenuation is observed with NP-PPi and PPi treatment, with an over 90% decrease in collagenase activity (Figure 5F) as compared to control. Although significant decreases in normalized collagenase levels with blank NP treatment was achieved at 8 h, this effect may be attributed to the observed increase in growth (Figure 5D) as no statistical differences in raw collagenase levels for *S. marcescens* exist in the presence of blank NPs at the 6 or 8-h time point (Figure 5E). Thus, the data in Figure 5 demonstrate that NP-PPi and equivalent PPi treatments are effective at suppressing collagenase activity of gram-negative pathogens (*P. aeruginosa* and *S. marcescens)* and that treatment with blank NP-PPi does not hinder their survival. In contrast to the observed responses observed with the gram-negative pathogens, blank NPs, NP-PPi, and free PPi were not as effective at attenuating collagenase levels of gram-positive *E. faecalis* (Figures 5H,I). In terms of normalized collagenase levels (Figure 5I), significant reductions in collagenase levels (~20%) are observed for the NP-PPi treatment group as compared to control. The exact reason for the enhancement in collagenase attenuation achieved with NP-PPi as compared to free PPi treatment is unknown. One possible explanation of these findings may be linked to quorum sensing, or to PEG interactions with the bacterial membrane. Nonetheless, these observations support the hypothesis of NP-PPi as an effective treatment for attenuation of collagenolytic activity, and most effective in the case of the gram-negative pathogens.

**Figure 6:**
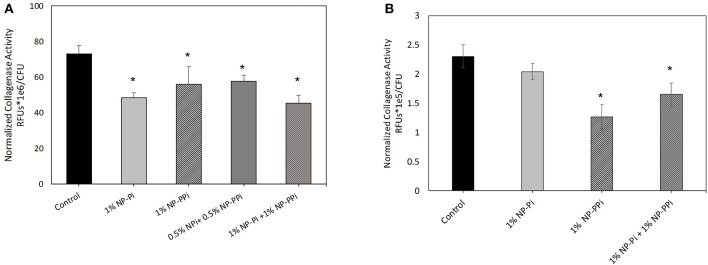
Effect of NP-Pi, NP-PPi, and NP-Pi + NP-PPi mixture on collagenase attenuation of **(A)** *E. faecalis* and **(B)** *P. aeruginosa*. Data reported in terms of collagenase activity (RFUs) normalized to growth (CFUs). Error bars indicate plus and minus the standard deviation. Asterisk {_*_} denotes significance vs. control with *p* < 0.005 considered statistically significant.

The inability of free PPi and consequently NP-PPi to attenuate collagenase activity of *E. faecalis* to the same extent as the gram-negative test pathogens is not entirely clear. It is known, however, that polyphosphate kinases (ppk1 and ppk2) are primarily responsible for synthesizing and hydrolyzing polyphosphates in bacteria. These enzymes, and high polyphosphate storage capacity have been confirmed in pathogens such as *P. aeruginosa* (Zhang et al., 2002; Racki et al., 2017), but recent studies have shown that the *Enterococcus* genus has a low genetic potential for accumulating and hydrolyzing polyphosphate (Breiland et al., 2018). Therefore, a possible explanation of our findings is that *E. faecalis*, does not have the capacity to readily break down PPi to phosphate to make it more readily bioavailable. Prior published studies indicate that oral administration of PPi does not promote healing in the surgically injured colon of mice intestinally inoculated with collagenolytic *E. faecalis* (pathogen-induced intestinal anastomotic leak) (Wiegerinck et al., 2018). Interestingly, a 20-mer of phosphate (Pi-20) covalently attached to a high molecular weight linear PEG block copolymer was found to suppress *in vitro* collagenolytic activity and to enhance anastomotic healing in mice intestinally inoculated with collagenolytic *E. faecalis* (Wiegerinck et al., 2018). These findings suggest that while replenishment of phosphate levels at the injured host-pathogen interaction site are crucial to healing, a single form of phosphate may not be universally effective at attenuating collagenase expression. This indicates that delivery of different forms of phosphate may be required to confer broad spectrum efficacy across collagenolytic pathogens. To address this hypothesis, we investigated the effects of NP-Pi and the combination treatment (NP-Pi+NP-PPi) on collagenase attenuation of *E. faecalis*. The data in Figure 6A indicate that the 1% NP-Pi + 1% NP-PPi combination yields the greatest attenuation of normalized *E. faecalis* collagenase production (38% decrease) as compared to 1% (w/vol) NP-Pi (34% decrease), 1% (w/vol) NP-PPi (23% decrease), and a combination of 0.5 % N-PPi + 0.5 % NP-Pi (22% decrease) from the no treatment control. Importantly, the 1% NP-Pi + 1% NP-PPi treatment significantly decreased *P. aeruginosa* collagenase production to a similar degree as NP-PPi alone. Finally, 1% NP-Pi did not reduce *P. aeruginosa* collagenase production (Figure 6B). Combined, the data in Figure 6 demonstrate that the combination treatment involving sustained delivery of both PPi and Pi is most effective in attenuating collagenase production of the tested gram-positive and gram-negative pathogens. These findings are important as they form the basis for testing the efficacy of our approach *in vivo* and to further optimize the phosphate nanoparticle combination treatment in animal models of intestinal injury and healing.

### Effect of NP-PPi Treatment on Biofilm Production

Biofilm is a distinct pathogenic phenotype which plays a prominent role in tissue colonization and drug resistance. Therefore, the *in vitro* efficacy of sustained delivery of phosphates on biofilm production was also investigated. In previous studies we have shown that free PPi significantly attenuates biofilm production of *P. aeruginosa* and *S. marcescens* (Hyoju et al., 2017), however, the efficacy of NP-Pi, NP-PPi, or combinations of the two, on biofilm attenuation has not been previously explored.

**Figure 7:**
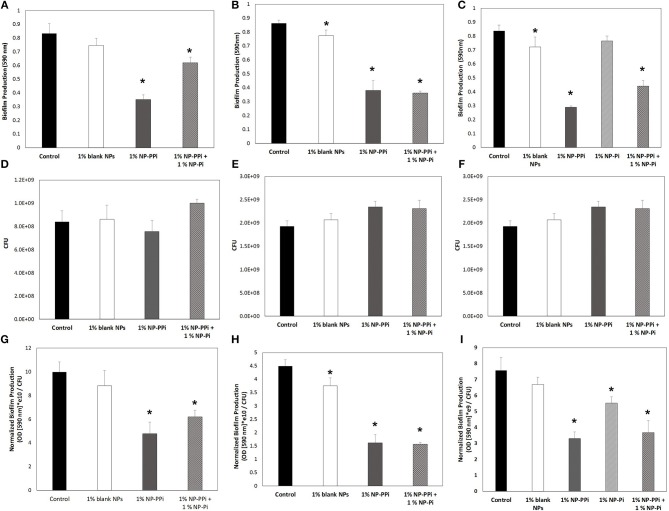
Effect of nanoparticle formulations on biofilm attenuation of the tested pathogens. Raw biofilm levels shown for **(A)** *P. aeruginosa*, **(B)** *S. marcescens, and* **(C)** *E. faecalis*. CFU values shown for **(D)** *P. aeruginosa*, **(E)** *S. marcescens, and* **(F)** *E. faecalis*. Normalized biofilm values shown for **(G)** *P. aeruginosa*, **(H)** *S. marcescens, and* **(I)** *E. faecalis*. Error bars indicate plus and minus the standard deviation. Asterisk {_*_} denotes significance vs. control with *p* < 0.05 considered statistically significant.

Based on our observed findings involving collagenase attenuation (Figures 5, 6), we investigated whether similar treatments were effective against biofilm production. The data in Figure 7 indicate that for all test pathogens the 1% NP-PPi and 1% NP-Pi + 1% NP-PPi treatments significantly reduce raw (Figures 7A–C) and normalized (Figures 7G–I) biofilm production as compared to the no treatment control. In the case of *P. aeruginosa*, the reduction in normalized biofilm from the control was found to be 52% with NP-PPi and 38% with the combination treatment (Figure 7G). For *S. marcescens* attenuation in normalized biofilm between the two phosphate treatments was found to be similar (64 and 65% reduction for NP-PPi and NP-Pi + NP-PPi, respectively, from the control). Interestingly, the greatest reduction in *E. faecalis* biofilm production occurred with NP-PPi (56% reduction) treatment, followed by the combination treatment (51% reduction) and NP-Pi (27% reduction) from the no treatment control (Figure 7I). These findings corroborate with those observed for collagenase attenuation, further validating our hypothesis that the NP-Pi + NP-PPi formulation provides broad spectrum treatment by attenuating collagenase and biofilm levels of gram-negative and gram-positive intestinal pathogens whose phenotype impairs healing.

## Conclusion

In summary, PEG hydrogel nanoparticles have been synthesized for sustained release of monophosphate (sodium monobasic phosphate) and polyphosphate (sodium hexametaphosphate). Our findings indicate that a combination treatment of NP-Pi and NP-PPi was most effective at attenuating collagenase and biofilm production in clinically relevant strains of gram-negative *P. aeruginosa* and *S. marcescens* and gram-positive *E. faecalis* expressing high collagenolytic activity. It is important to note that the nanoparticle formulations did not display bacteriostatic effects across the gram-negative and gram-positive pathogens tested, which indicates their potential as an effective drug delivery approach to target virulence without eradicating the normal microbiome which is beneficial to healing. A variety of other phenotypes, not investigated in this study, contribute to bacterial virulence, including excessive production of elastases, proteases, alginates, rhamnolipids, and hemolysins. Our future efforts will focus on determining whether our proposed treatment using phosphate and/or polyphosphates and combinations thereof are also capable of attenuating these pathogenic phenotypes. Future studies will also utilize reporter strains of the test pathogens to quantify the efficacy of the proposed treatment on quorum sensing suppression. Finally, our current efforts focus on evaluating the mucoadhesiveness of the proposed Pi and PPi nanoparticle formulations on explants of intestinal injury and their pre-clinical efficacy in accelerating healing in murine model of intestinal injury.

## Data Availability

All datasets generated for this study are included in the manuscript and/or the Supplementary Files.

## Author Contributions

DN, OZ, SHH, SKH, FT, JA, and GP contributed conception and design of the study. DN, MP, SHH performed the experiments. DN and MP performed the statistical analysis. DN wrote the first draft of the manuscript. DN, MP, FB, and GP wrote sections of the manuscript. All authors contributed to manuscript revision, read, and approved the submitted version.

### Conflict of Interest Statement

The authors declare that the research was conducted in the absence of any commercial or financial relationships that could be construed as a potential conflict of interest.
