# Supplementary material for: Sustained Release of Phosphates From Hydrogel Nanoparticles Suppresses Bacterial Collagenase and Biofilm Formation in vitro
Source: Front Bioeng Biotechnol. 2019 Jun 26;7:153. doi: 10.3389/fbioe.2019.00153 (PMC6607000; doi:10.3389/fbioe.2019.00153)
Supplement: Supplementary file 1 [file Data_Sheet_1.PDF]

Supplementary Material

Sustained Release of Phosphates from Hydrogel Nanoparticles Suppresses Bacterial Collagenase and Biofilm Formation *In Vitro*

Dylan Nichols,[1] Marja B. Pimentel,[1] Fernando T.P. Borges,[2] Sanjiv K. Hyoju,[3] Fouad Teymour,[2] Seok Hoon Hong,[2] Olga Y. Zaborina,[3] John C. Alverdy,[3] and Georgia Papavasiliou[1*]

1. Department of Biomedical Engineering, Illinois Institute of Technology, Chicago, IL
2. Department of Chemical and Biological Engineering, Illinois Institute of Technology, Chicago, IL
3. Department of Surgery, University of Chicago, Chicago, IL

*** Correspondence:** Dr. Georgia Papavasiliou - papavasiliou@iit.edu - 312.567.5959

Supplementary Data

A. Luminescence versus colony forming units (CFUs) standard curves

Since the presence of nanoparticles interfere with optical density measurements, bioluminescent strains of bacteria were used to measure growth. Highly collagenolytic gram-negative bioluminescent strains were either constructed (*S. marcescens)* in our laboratories or purchased from commercially available vendors (*P. aeruginosa*). Since each bacterial strain differs in its strength of luminescence, the measured luminescence from each pathogen corresponds to a different bacterial density. To address this issue, a standard curve was created for each pathogen comparing luminescence to CFUs and linear regression was used to convert luminescence measurements of either *P. aeruginosa* (Figure S1) or *S. marcescens* (Figure S2) to 'effective' CFU values.

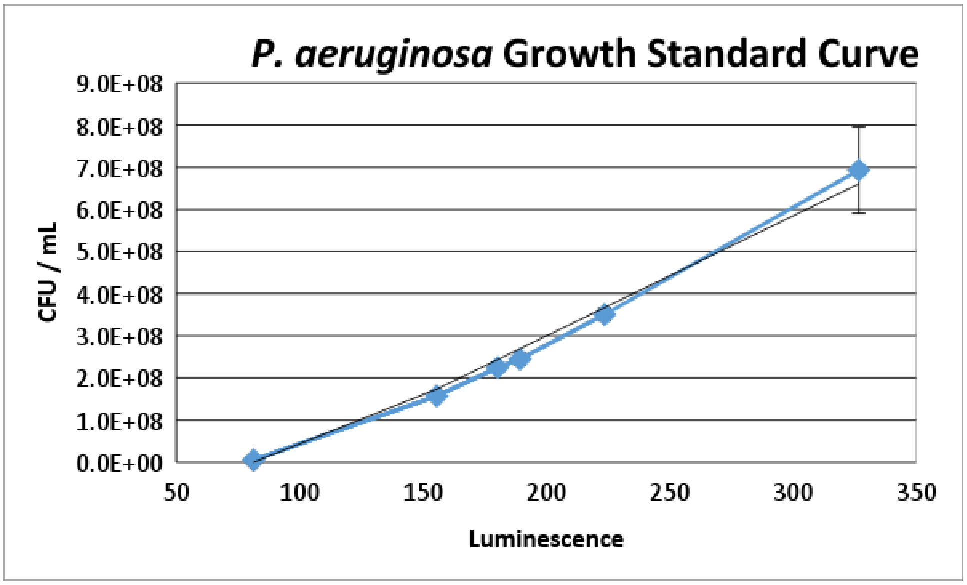

Figure S1: Standard curve for *P. aeruginosa* luminescence versus CFUs. Linear regression yields a coefficient of determination of $r^2 = 0.98$.

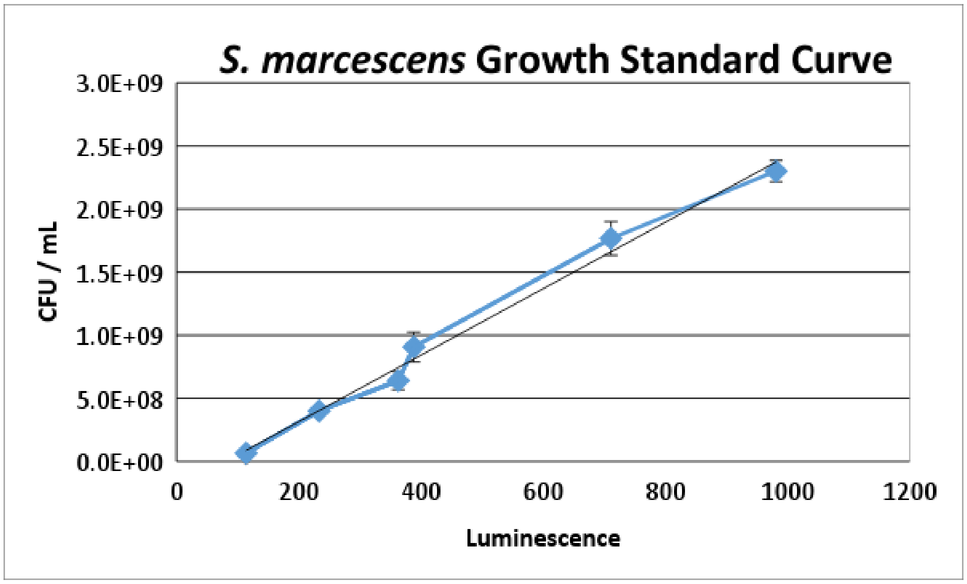

Figure S2: Standard curve for *S. marcescens* luminescence versus CFUs. Linear regression yields a coefficient of determination of r^2=0.98.
